# Interaction of Antimicrobial Peptide Ponericin W1, Thanatin, and Mastatopara-S with *Geotrichum citri-aurantii* Genomic DNA

**DOI:** 10.3390/foods10081919

**Published:** 2021-08-18

**Authors:** Hongyan Zhang, Sha Liu, Xindan Li, Wenjun Wang, Lili Deng, Kaifang Zeng

**Affiliations:** 1 College of Food Science, Southwest University, Chongqing 400715, China; 18789420684@163.com (H.Z.); a1499399857@163.com (S.L.); 18227589580@163.com (X.L.); wangwenjun_w@163.com (W.W.); denglili_316@163.com (L.D.); 2 Food Storage and Logistics Research Center, Southwest University, Chongqing 400715, China

**Keywords:** *Geotrichum citri-aurantii*, antimicrobial peptide, location, DNA binding

## Abstract

Antimicrobial peptides of mastatopara-S (M-S), thanatin, and ponericin W1(P W1) were able to disrupt the membrane integrity and alter the morphology of the hyphae of *Geotrichum citri-aurantii* and then reduced the sour rot of citrus fruit. In order to understand the mechanisms of thanatin, P W1 and M-S other than membrane disruption, the interaction betwixt the peptides and *G. citri-aurantii* DNA were investigated in this research. The laser confocal microscopy found that P W1, thanatin, and M-S could penetrate the cell membrane. Gel retardation assay demonstrated that P W1, thanatin, and M-S could bind to the *G. citri-aurantii* genomic DNA in vitro. UV-visible spectra and fluorescence spectra analysis further confirmed that the peptides can bind to the DNA, and then insert into the base pairs in the DNA helix, followed by wrecking the double-helix structure. In addition, M-S, thanatin, and P W1 can suppress the synthesis of DNA and RNA of *G. citri-aurantii*.

## 1. Introduction

Among the common postharvest invasive diseases of citrus, green mold and blue mold are the diseases with the highest incidence, causing fruit rot rates generally ranging from 10% to 30%, resulting in serious citrus industry losses [1]. The situation of sour rot caused by *Geotrichum citri-aurantii* is inferior to blue mold and green mold, and which is becoming increasingly severe due to the lack of effective control methods [2]. Currently, common fungicides such as benzimidazoles, imidazoles, biguanides, and others were used for controlling postharvest disease of citrus [3,4]. For the control of sour rot of citrus, the fungicides mentioned above were ineffective against *G. citri-aurantii*, except for Bellkute, but the large-scaled and yearly application of which has led to an increasingly resistance to the fungicide [5]. The residual of chemical fungicides have made the quest for safer and more effective green fungicides increasingly urgent [6], and the use of antimicrobial peptides to control postharvest sour rot of citrus fruits was one of the effective alternatives to chemical fungicides [2].

Antimicrobial peptides (AMPs) are short-sequence peptides (less than 50 amino acid residues) produced by plants or animals, providing broad-spectrum inhibition towards parasites, fungi, viruses, and bacteria [7]. Due to their strong antimicrobial ability, which was only slightly weaker than that of fungicides [8], environmental friendly, low level of antibiotic resistance and capacity to be produced on a massive scale by bioreactors or genetically engineered bacteria, AMPs are regarded as potential substitute to conventional fungicides for controlling postharvest diseases of fruits and vegetables [9]. For the control of postharvest diseases of fruits and vegetables, as new biological control agents, antifungal peptides have been investigated. At present, researchers have discovered many peptides that showed outstanding defense activity against postharvest pathogens. For example, a novel peptide CgPep33, isolated from enzymatic hydrolysates obtained from the Pacific oyster (*Crassostrea gigas*) has high inhibitory activity against *Botrytis cinerea* growth and can effectively reduce the disease incidence of gray mold on postharvest strawberry fruit [10]. Fengycin, the production of *Bacillus subtilis* CPA-8, can provide effective control of peach brown rot [11]. Additionally, hybrid undecapeptide BP22 is active against *Penicillium expansum*, the cause of penicillium rot of apples [8]. ε-Poly-l-lysine (ε-PL) possessed antibacterial activity against *Alternaria alternata* in vivo and in vitro, which is a natural antimicrobial poly-cationic peptide, and ε-PL lead to morphological changes, lack of membrane integrity and oxidative stress of *A. alternata* [12].

Antifungal peptides are typical short chain of amphipathic cation, the defense mechanism of which may be a disruption of the cell membrane of the target fungal, leading to the increase in membrane permeability. However, it has been proved that the increase in cell membrane permeability does not necessarily lead to cell death [13]. An increasing amount of researchers believe that peptides have other non-membrane permeability mechanisms rather than membrane permeability. In the past decade, many researches have shown that peptides can bind to intracellular targets, for instance, binding to nucleic acid materials [14], inhibiting nucleic acid or protein synthesis [15], and inhibiting enzyme activity [16].

The three peptides we used were essentially insect antimicrobial peptides. Mastoparan-S(M-S), thanatin, and ponericin W1(P W1) were isolated from *Sphodromantis viridis*, *Podisus maculiventris* and *Pachycondyla goeldii*, respectively, and exhibited antibacterial activity towards most Gram-positive and Gram-negative bacteria as well as fungi [17,18,19]. In our previous study, we have found that M-S, thanatin, and P W1 disrupted the membrane integrity and altered the morphology of the hyphae of *G.citri-aurantii* and then reduced the sour rot of citrus fruit [2]. However, it was not clear whether these peptides could penetrate the cell membrane of *G.citri-aurantii* and interact with *G. citri-aurantii* DNA. Thus, the purpose of this research was to further explore the mechanisms of M-S, thanatin, and P W1 against *G. citri-aurantii* mainly related to genomic DNA.

## 2. Materials and Methods

### 2.1. Materials

Peptide, mastatopara-S (M-S) thanatin, and ponericin W1(P W1) were prepared as described by Liu et al. [2]. The peptides labeled with the green fluorescent dye fluorescein isothiocyanate (FITC) were procured from GenScript Corporation (Nanjing, China) with >95% purity. Peptides were chemically prepared using N-(9-fluorenyl) methoxycarbonvl (Fmoc) chemistry by solid-phase methods. The respective peptides were formulated as stock solutions at a concentration of 1 mmol L^−1^ in sterile distilled water and stored at −40 °C. The pathogen of *G. citri-aurantii* was isolated and identified internally by our laboratory [2] (College of Food Science, Southwest University, Chongqing, China).

### 2.2. Pathogen Culture

Potato dextrose agar (PDA) was used for incubating the strain of *G. citri-aurantii* at 25 °C. Conidia from 7-day-old PDA cultures were harvested and were resuspended in sterile distilled water. A hemocytometer was used to count the conidial suspension and the concentration was regulated to the desired concentration (1 × 10^6^ CFU mL^−1^).

### 2.3. Determining the Location of Peptides Using FITC Labelling

Marking of the n-terminus of M-S, thanatin, and P W1 with the green fluorescent dye FITC under Leica TSC-SP2 confocal microscope (Nikon Eclipse 90i) to identify the location of the peptide inside *G. citri-aurantii* cells. The experiment was performed in accordance with Omardien et al. [20] with some slight modifications. The *G. citri-aurantii* conidial suspension (1 × 10^6^ CFU mL^−1^) was processed with FITC-peptides for 2 h at 25 °C, and washed twice with PBS solution (10 mmol L^−1^, pH 7.5) to discard remaining labeled peptide. Afterwards, PI staining solution at a final concentration of 10 μg mL^−1^ was applied to the treated conidial suspension and stained for 5 min protected from light. A drop of the mixture was pipetted onto a slide, overlayed with a coverslip. The confocal laser-scanning microscopy (Nikon Eclipse 90i) was used for acquisition of the fluorescent images (For PI detection: 535 nm was set as the excitation wavelength and the detection wavelength at 615 nm; for FITC-Peptide detection: the excitation wavelength was set at 488 nm and the detection wavelength at 520 nm).

### 2.4. Preparation of G. citri-aurantii Genomic DNA

Extraction of genomic DNA from *G. citri-aurantii* by the Sodium dodecyl sulfate (SDS) method was referred to Liu et al. [21] with appropriate adaptations, and the steps were as follows.

(1) Take about 0.1 g of fresh fungal hyphae, grind them to powder in liquid nitrogen and put in a 1.5 mL Eppendorf tube.

(2) Add 500 μL of DNA extraction solution, shake gently and mix well, and then place in water bath at 65 °C for 10 min.

(3) Add 200 μL of 3 mol L^−1^ NaAc (pH 5.2), mix well, and then place in ice for 20–30 min. Centrifuge at 13,000 rpm for 10 min at 4 °C and transfer the supernatant to a new Eppendorf tube.

(4) Equal volumes of phenol: chloroform (1:1) mixture was added, shake vigorously for several times, then centrifuge to obtain the supernatant, and repeat this procedure for one more time.

(5) Shift the above supernatant to a new Eppendorf tube, add 0.6 times the volume of isopropanol (pre-chilled at −20 °C), mix by shaking up and down lightly and place in the refrigerator at −20 °C for 30 min.

(6) Centrifuge at 13,000 rpm for 15 min at 4 °C, the supernatant was discarded, wash the precipitate 2–3 times with 75% anhydrous ethanol and air dried to evaporate the anhydrous ethanol.

(7) Dissolve in 20 μL of Tris-EDTA buffer solution (TE buffer) and store in a −20 °C refrigerator.

(8) The purity of the extracted genomic DNA was assessed by the optical density ratio of 260 nm and 280 nm (OD260/OD280 = 1.91) [22]. The concentration of DNA was measured by the determination of the absorbance at 260 nm (BioTek Instruments, Inc., Vermont, VT, USA) at room temperature.

### 2.5. Gel Retardation Assay for Binding of Antimicrobial Peptides to G. citri-aurantii Genomic DNA

The gel retardation assay for the binding of antimicrobial peptides to DNA was conducted as described before with some modifications [2,23]. The extracted *G. citri-aurantii* genomic DNA was solubilized in TE buffer, and added an equivalent volumes of antimicrobial peptide solution (1:1) to a final concentration of 1.56, 3.12, 6.25, 12.5, 25, 50, and 100 μmol L^−1^. A control group was formed without the addition of antimicrobial peptide. The mixture was mixed evenly and incubated for 1 h at room temperature. Then, 1 μL of 6× loading buffer was added to 5 μL of the treated solution and examined by electrophoresis using 0.8% agarose gel in 1× TAE buffer, and operated at a voltage of 110 V for 30 min. Tanon 4100 (Tanon Science and Technology Co., Ltd., Shanghai, China) was applied to view the gel images and photographed for analysis.

### 2.6. UV-Visible Spectra

The assay was referenced to the technique of Tang et al. [14] with appropriate modifications. The *G. citri-aurantii* genomic DNA was diluted to 50 μg mL^−1^ with sterile water, and mixed with a gradient concentration of peptides at 1:1 (*v*/*v*) for 30 min in the darkness at room temperature. The final peptides concentrations were set as 0, 1.56, 3.12, 6.25, 12.5, 25, 50 and 100 μmol L^−1^. The spectra of the mixture were determined in the range of 230–400 nm by Multiskan Spectrum microplate spectrophotometer (BioTek Instruments, Inc., Vermont, VT, USA).

### 2.7. Antimicrobial Peptides Competitively Bind DNA with Ethylene Diamine (EB)

The measurements were performed according to the method of Li et al. [24] with suitable modifications. *G. citri-aurantii* genomic DNA was dissolved in 1 mL sterile distilled water to a final concentration of 50 μg mL^−1^. Then, mixed with 15 μL EB solution at a concentration of 100 μg mL^−1^ and subsequently incubated for10 min in the darkness. An equal volume of different concentrations of peptides were added into the mixture solution for 30 min in the darkness at room temperature, and the final peptides concentrations were at 0, 1.56, 3.12, 6.25, 12.5, 25, 50 and 100 μmol L^−1^. The samples were detected while the fluorescence excitation wavelength was 535 nm, and the fluorescence emission wavelength range from 560 to 700 nm, the sampling interval was 1 nm.

### 2.8. Influence of Antimicrobial Peptides on DNA and RNA Synthesis in G. citri-aurantii

4′,6-diamidino-2-phenylindole (DAPI) staining method was used to quantify the nucleic acids of *G. citri-aurantii*, the experiment was done by drawing on the method of Wang et al. [25] with adjustments as appropriate. The *G. citri-aurantii* conidial suspension at a concentration of 1 × 10^6^ CFU mL^−1^ was treated with peptides in shaker (150 r min^−1^) at 25 °C for 0.5, 2, 4, 6 and 12 h. Then, stained with 15 μg mL^−1^ DAPI under controlled darkness for 10 min. The fluorescence density of nucleic acid was individually determined with a Multiskan Spectrum microplate spectrophotometer (BioTek Instruments, Inc., Vermont, VT, USA). The excitation wavelength of DNA was 364 nm and RNA was 400 nm, and the emission wavelengths were always 460 nm. The reference control group was the same solution but without peptides. Each treatment was replicated 3 times.

### 2.9. Statistical Analysis

Every treatment was conducted in triplicate. One-way analysis of variance (ANOVA) followed by Dun-can’s multiple-range test (SPSS Inc., PASW Statistics for Windows, Version 16.0, Chicago, IL, USA) were used for analyze all data. Statistical significance was accepted as *p* < 0.05.

## 3. Results

### 3.1. Localization of Peptides in G. citri-aurantii Conidia Cells

The cellular location of peptides in the *G. citri-aurantii* cells was measured using confocal laser-scanning microscopy. *G. citri-aurantii* conidia cells were processed with 64 μmol L^−1^ of FITC-labelled M-S, 64 μmol L^−1^ of FITC-labelled thanatin and 32 μmol L^−1^ of FITC-labelled P W1, respectively. Cells treated with FITC-labeled antimicrobial peptide showed green fluorescence if there were antimicrobial peptide remaining in the cells, and cells treated with PI staining showed red fluorescence if any PI remained in the cells, while the cells showed yellow or orange fluorescence in the merged images if both FITC-labeled antimicrobial peptide and PI remained in the cells after treatment.

**Figure 1:**
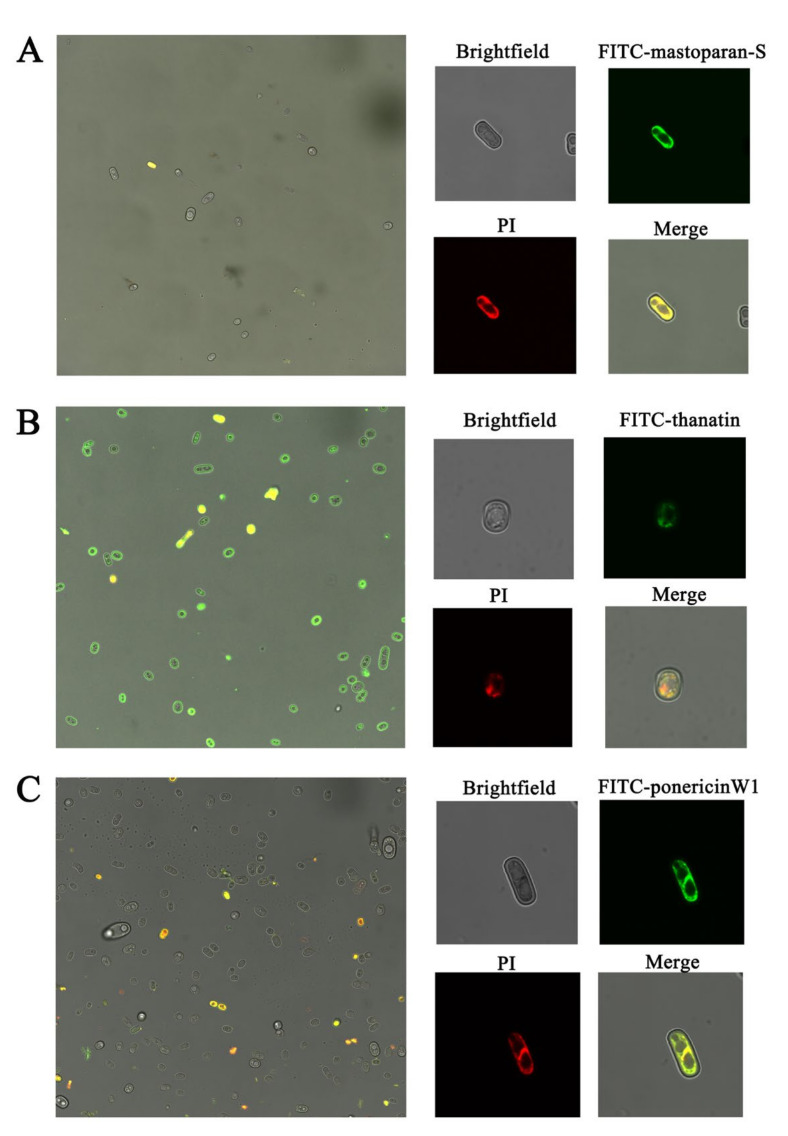
Confocal fluorescence microscopic images of *G. citri-aurantii* cells. *G. citri-aurantii* conidia cells were processed with 64 μmol L^−1^ of FITC-labelled M-S (**A**), 64 μmol L^−1^ of FITC-labelled thanatin (**B**) and 32 μmol L^−1^ of FITC-labelled P W1 (**C**) at 25 °C for 2 h. FITC-labelled peptides go through the cell membrane and gather inside cytoplasm. The figure on the **left** were the merged images of bright field and fluorescence images. Bright filed, fluorescence and merge images of each treatment are shown on the **right** side.

As shown in Figure 1A, neither green fluorescence nor red fluorescence was observed in the majority of conidia cells, only a minimal number of cells showed yellow fluorescence, suggesting that FITC-labelled M-S could penetrate into the intracellular of a few cells during the limited treatment time. As Figure 1B reveals, nearly all cells have fluorescence coloring, green fluorescence appeared on the edge of most conidia cells, indicating the FITC-labelled thanatin gathered on the surface of the cell membrane. The green fluorescence also appeared inside the cells, a handful of cells have residues of both FITC-labelled thanatin and PI, suggesting the FITC-labelled thanatin was also able to enter the cell membrane and gathered in the cytoplasm of the *G. citri-aurantii* conidia cells after treatment for 2 h. As displayed in Figure 1C, some cells in the merged image appear orange fluorescence, and both FITC- labelled P W1 and PI can traverse the cell membrane and gather inside the cell.

### 3.2. Gel Retardation Assay

**Figure 2:**
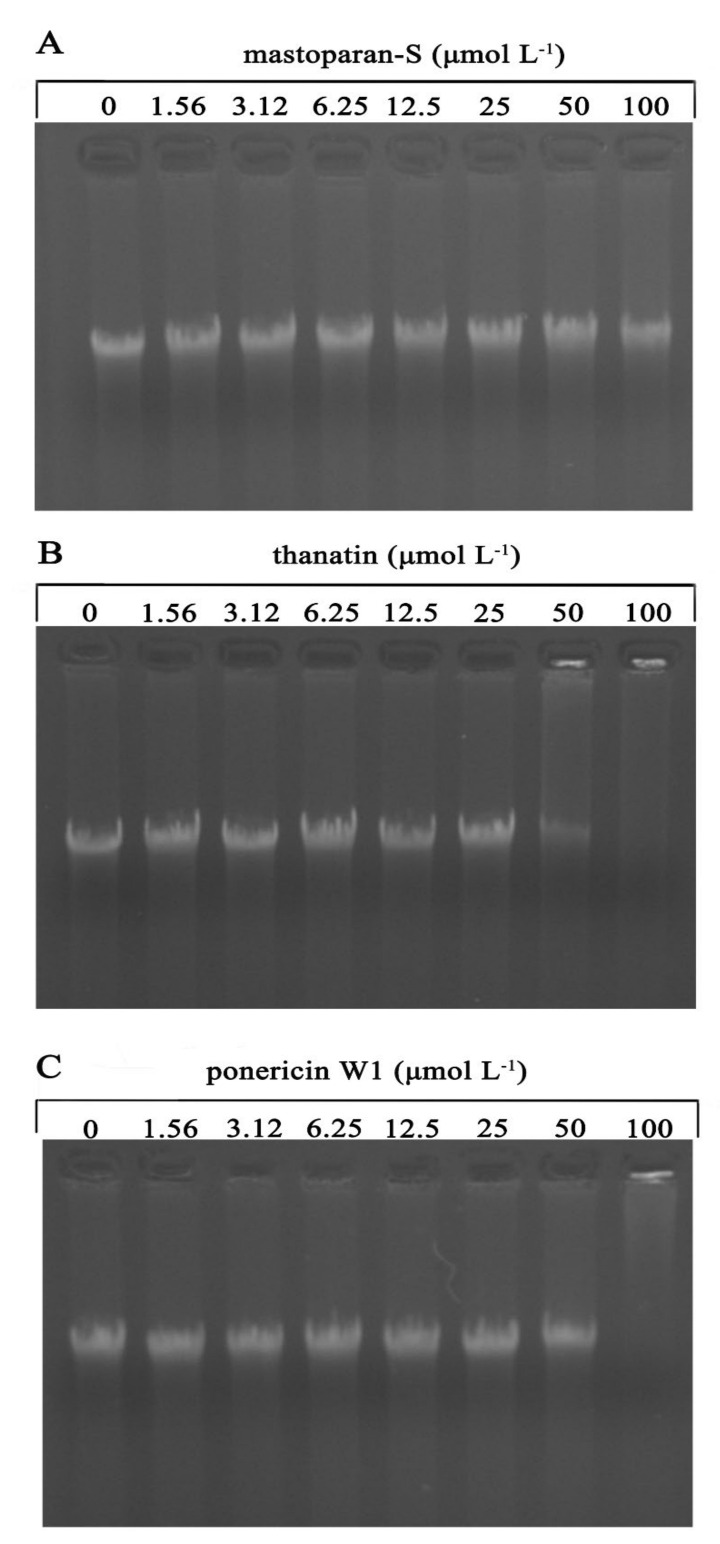
Gel retardation assay of the binding of peptides with *G. citri-aurantii* genomic DNA. No peptide addition (0) or different concentrations of M-S (**A**), thanatin (**B**) and P W1 (**C**) were mixed with *G. citri-aurantii* DNA by 1:1 (*v*/*v*) for 1 h at room temperature and analyzed by gel retardation experiment.

The capacity of binding to DNA of these three peptides were investigated by gel retardation experiment. The DNA of *G. citri-aurantii* which was treated with increasing amounts of peptides (1.56, 3.12, 6.25, 12.5, 25, 50 and 100 μmol L^−1^) was depicted in Figure 2. As displayed in Figure 2A, M-S has no effect on the migration of DNA and no DNA remained in the wells, which implying that M-S failed to bind with *G. citri-aurantii* genomic DNA at the experimental concentration range, whereas both P W1 and thanatin could varyingly bind with *G. citri-aurantii* genomic DNA. As shown in Figure 2B, upon incubation of DNA with 50 μmol L^−1^ of thanatin, DNA was partly remained in the well, the gel grown darker, which mean that a portion of thanatin was binding with the DNA at this concentration. When an increase in its concentration to 100 µmol L^−1^, the DNA disappeared, reflecting thanatin completely binding with the DNA at this concentration. The gel retardation of P W1 was presented in Figure 2C. When the final concentration was 100 µmol L^−1^, the DNA was completely stagnated in the wells and the genomic DNA vanished. The result indicating that P W1 and thanatin showed higher ability to bind with *G. citri-aurantii* genomic DNA than M-S.

### 3.3. UV-Visible Spectra

The UV-Vis absorption spectra provided important insights into effect of bindings of small molecules with the DNA [26]. Small molecules can interact with DNA by the way of intercalative association through planar, heteroaromatic moiety slides with the DNA base pairs. This binding commonly results in hypochromism on the UV-Vis absorption spectra, the more significant the hypochromism, the stronger the influence. When the double helix structure of DNA was destroyed by small molecules, it shows an hyperchromicity, likewise, the more pronounced the hyperchromicity, the heavier the damage [27]. Therefore, the binding mode of the peptides with the DNA of *G. citri-aurantii* can be determined by the variation of the UV spectrum.

**Figure 3:**
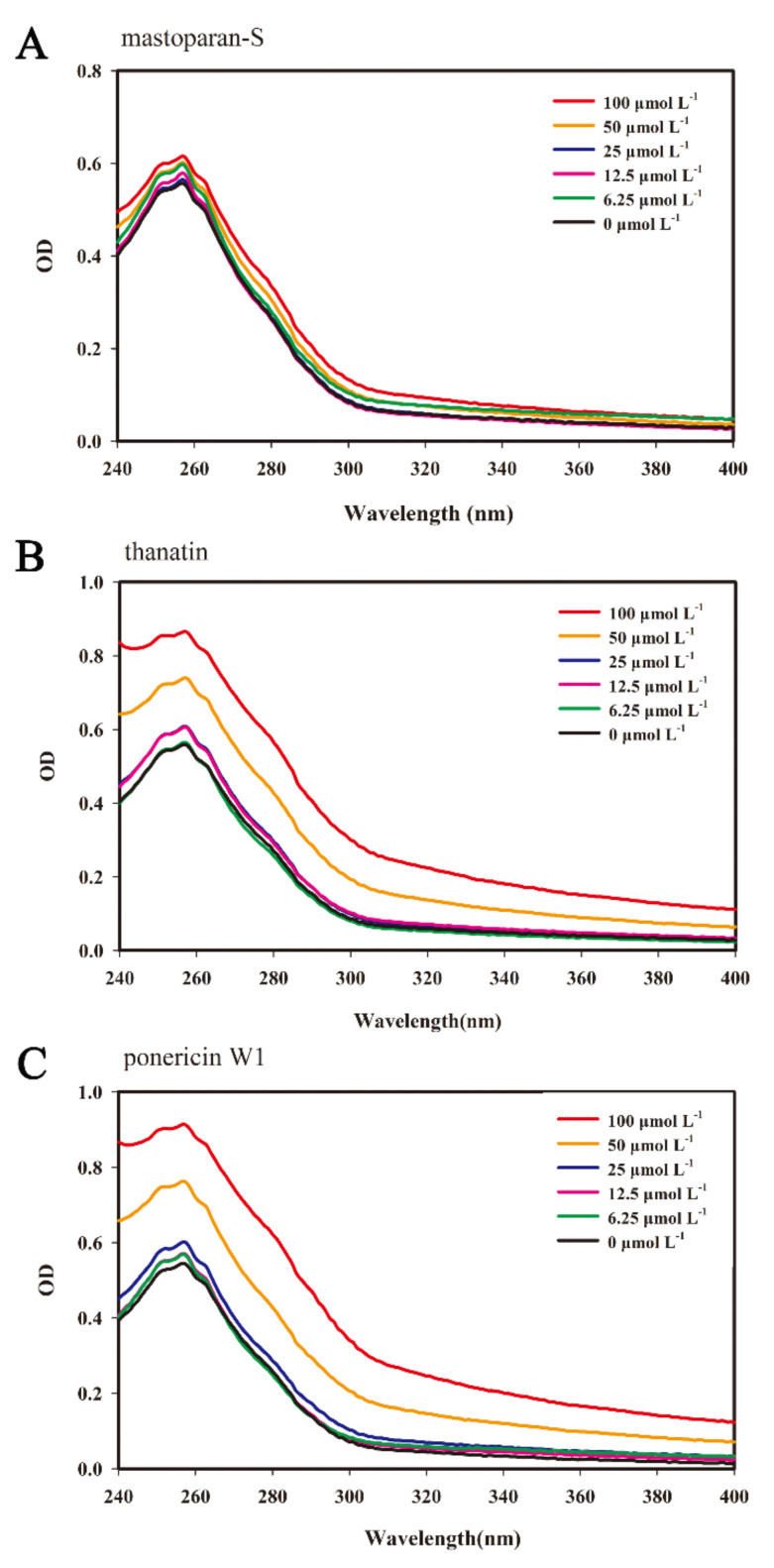
Ultraviolet spectrum of the interaction of M-S (**A**), thanatin (**B**) and P W1 (**C**) with *G. citri-aurantii* genomic DNA. Increasing concentrations of peptides (6.25, 12.5, 25, 50 and 100 µmol L^−1^) was added to 50 μL mL^−1^ of DNA by 1:1 (*v*/*v*) for 30 min. Ultraviolet spectra were conducted while the wavelengths series from 230 to 400 nm.

As displayed in Figure 3, the maximum peak of DNA was located at 257 nm, and there was no spectral shift after treatment with peptides. However, by increasing peptides concentration, the intensity of the maximum peak of DNA were obviously increased, suggesting the double helix structure of DNA may be destroyed by peptides to varying degrees. The maximum absorbance value of DNA solution without peptides was 0.545. M-S induced a weaker hyperchromicity, with a maximum absorbance value of 0.598 for DNA at the concentration of 6.25 µmol L^−1^, an elevation of 9.72% compared to the control. As the concentration of M-S increased to 100 µmol L^−1^, the maximum absorbance value of DNA was 0.616, which was 13.02% higher than the control (Figure 3A). The level of hyperchromicity induced by P W1 and thanatin were relatively higher, with the maximum absorbance values of 0.602 and 0.609 for DNA at concentrations of 25 µmol L^−1^, respectively, which were 10.46% and 11.74% elevated as compared to the control, the effect of this concentration were similar to that of M-S at 100 µmol L^−1^. When the concentration of P W1 and thanatin was increased to 100 µmol L^−1^, the maximum absorbance values of DNA were 0.914 and 0.866, respectively, which were 67.71% and 62.57% higher over the control (Figure 3B,C). Moreover, the hyperchromicity induced by M-S (Figure 3A) was weaker than that by P W1 (Figure 3C) and thanatin (Figure 3B). These results indicated that M-S, thanatin, and P W1 may destroy the double helix structure of DNA when treated at high concentration of peptides, possibly caused by binding with *G. citri-aurantii* genomic DNA.

### 3.4. Competitive Binding of Peptides and EB with G. citri-aurantii DNA

Ethidium bromide (EB) is a well-known fluorescence probe that showed high sensitivity and excellent selectivity. The fluorescence of free EB is weak, however, the fluorescence intensity can be intensified by binding to DNA nonspecifically, because of intercalation between adjacent base pairs within the double helical structure of DNA [24]. This enhanced fluorescence can be diminished while other small molecules existing in coexistence EB, which commonly be applied to identify the binding mode of peptides to DNA while EB binds originally to DNA by intercalation [28]. Thus, the pattern of M-S, thanatin, and P W1 were further elucidated by a competition binding experiment of peptides and EB with *G. citri-aurantii* DNA.

**Figure 4:**
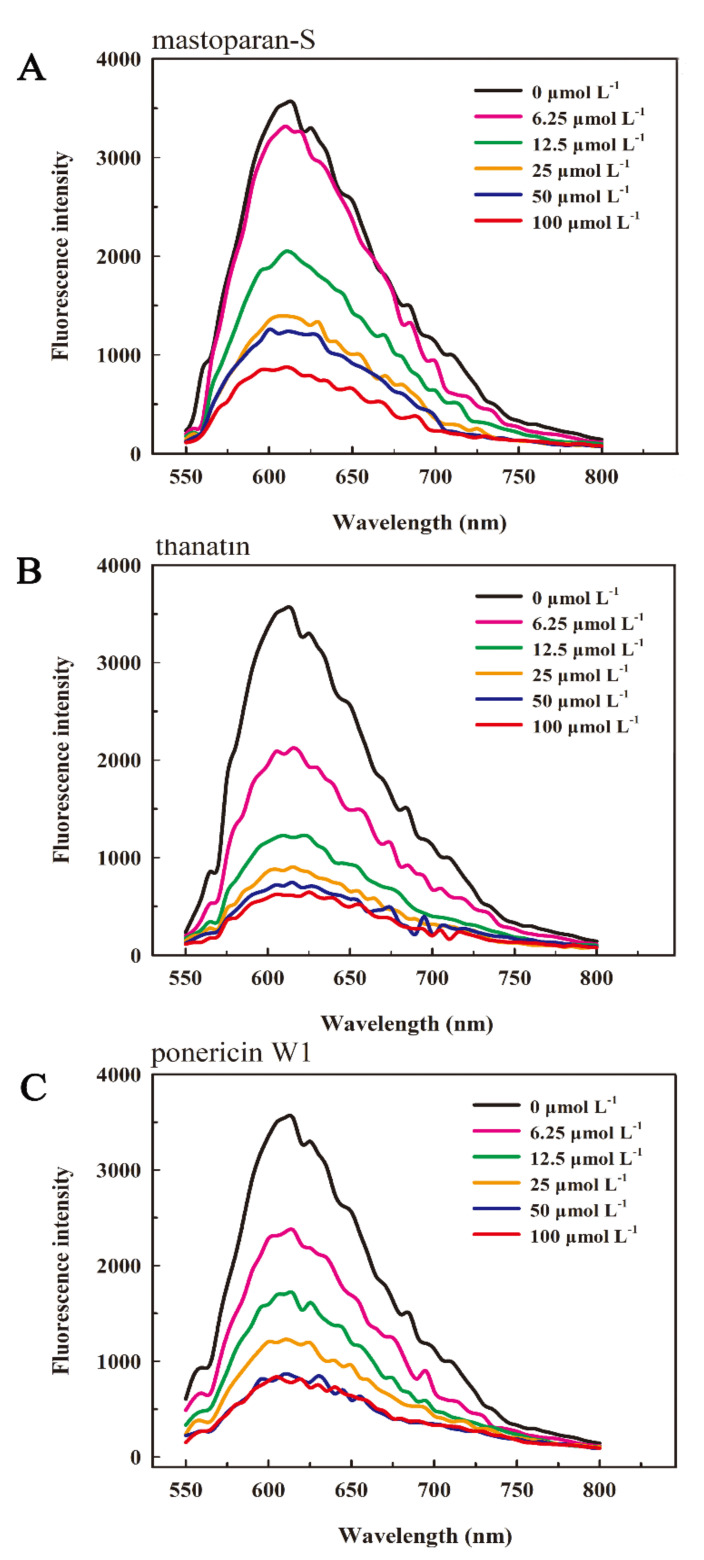
Competitive binding of M-S (**A**), thanatin (**B**) and P W1 (**C**) with *G. citri-aurantii* genomic DNA. Increasing concentrations of peptides (6.25, 12.5, 25, 50 and 100 µmol L^−1^) was added to 50 μL mL^−1^ of *G. citri-aurantii* DNA by 1:1 (*v*/*v*) for 30 min. The fluorescence spectra were documented from 560 to 700 nm (Ex = 535 nm).

The fluorescence spectra of EB-DNA mixture after addition of series concentrations of peptides were depicted in Figure 4. The fluorescence intensity of the mixture diminished in varying degrees with the addition of series concentrations of peptides, however, the location of the emission peaks remained unaltered, suggesting that the addition of the peptides did not modify the microenvironment around the EB-DNA, but could competitively bind to DNA with EB, and the free EB lowered the fluorescence intensity.

By Figure 4A it can be noticed that with the increasing of the concentration of M-S, the fluorescence quenching was observed, indicating that M-S competed with EB in binding to DNA. A stronger fluorescence quenching was observed when adding P W1 (Figure 4C) and thanatin (Figure 4B) into the EB-DNA mixture. When the concentration was 6.25 µmol L^−1^, the fluorescence intensity of EB-DNA dropped from 3549 with no peptide to 2370, 2123 and 3316, respectively, which containing M-S, thanatin, and P W1. When the concentration was extended to 100 µmol L^−1^, the fluorescence intensity of these individual EB-DNA declined to 831, 644 and 929; suggested that M-S, thanatin, and P W1 could compete with EB in binding with *G. citri-aurantii* DNA and partial EB molecules inserted into DNA base pairs were substituted by peptides. Moreover, the binding affinity of P W1 and thanatin was stronger than that of M-S.

### 3.5. Effect of Peptides on G. citri-aurantii Nucleic Acid Synthesis

**Figure 5:**
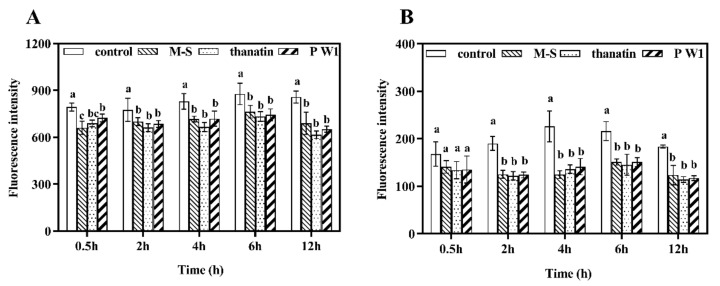
Impact of peptides on the synthesis of nucleic acid in *G. citri-aurantii* for 12 h. (**A**) Changes in DNA effected by control group (blank bar), P W1 (thin line bar), thanatin (spot bar) and M-S (thick line bar). (**B**) Changes in RNA effected by control group (blank bar), P W1 (thin line bar), thanatin (spot bar) and M-S (thick line bar). The data are showed as means ± SD. Different letters refer to difference among treatments based on Ducan analysis was significant (*p* < 0.05).

DAPI is a fluorescence dye which can bind with nucleic acid. Its fluorescence increases with the increment in the quantity of nucleic acids [25]. Thus, DAPI staining was applied to investigate the changes in DNA and RNA upon the application of M-S, thanatin, and P W1 on *G. citri-aurantii.* As given in Figure 5, the DNA synthesis was significantly decreased by 23.97%, 28.17% and 19.58% after treated with M-S, thanatin, and P W1 for 12 h, respectively. Additionally, the RNA synthesis was also significantly reduced by 36.30%, 37.75% and 32.67% after treated with M-S, thanatin, and P W1 for 12 h, respectively. The results indicated that M-S, thanatin, and P W1 could prevent nucleic acid synthesis of *G. citri-aurantii*, inducing a decrease in the nucleic acid content.

## 4. Discussion

Peptides labeled with the fluorescent dye are usually used to investigate the localization of antimicrobial peptides in cells or the process of interacting with cell membrane by a laser confocal laser scanning microscope [29]. In the previous study, we found that M-S, thanatin, and P W1 can change the morphology and disrupt the membrane integrity of the *G. citri-aurantii* hyphae. In order to study whether these peptides can go through the cell membrane, we determine the location of the peptide inside the *G. citri-aurantii* cells by confocal microscope method. The consequences revealed that M-S, thanatin, and P W1 can go through the cell membrane and concentrated in the cytoplasm of the *G. citri-aurantii* cells, indicating that these peptides were possibly to interact with intercellular molecules of microorganisms. The Shai-Matsuzaki-Huang model suspected that peptides initially bind to the surface of membrane and then penetrate into the cellular owing to their amphipathic structure, leading to lipid chain associations break up and form transient pores [30]. The mechanisms might be concentration related, as it has been found that peptide under a critical concentration can induce a disruption of the membrane, peptide passage preserved the integrity of the membrane, which was only temporarily disrupted [31]. Although this model cannot fully explain the penetration of M-S, thanatin, and P W1 into *G. citri-aurantii* cell, there may be some similar models for these three peptides, which need to be further studied.

The interaction between cationic antimicrobial peptide and anionic biofilm has always been a hot research topic in antibacterial peptides, with the purpose to improve its antibacterial activity and stability, and to explore the possibility of antibacterial peptides in practical application. In the past decade, increasing evidence has been found that many AMPs have intracellular targets, such as DNA or RNA. Buforin II, isolated from the stomach tissue of Asian toad, has been discovered to be able to bind with nucleic acids of cells after penetrated the cell membranes, leading to rapid cell death [32]. Indolicidin, which has been described to suppress DNA synthesis and induce filamentation of bacteria, can bind to DNA in gel retardation and fluorescence quenching experiments [33]. In this stidy, P W1 and thanatin showed the ability to bind with *G. citri-aurantii* genomic DNA in gel retardation, but a much weaker DNA binding ability was found in M-S treated group.

Small molecules can interact with DNA through an intercalative association in which a planar, heteroaromatic moiety slides between the DNA base pairs. This binding commonly results in hypochromism on the UV-Vis absorption spectra. When the double helix structure of DNA are destroyed by small molecules, it shows an hyperchromicity [27]. For example, Liu et al. [34] found that Cecropin-XJ works on Staphylococcus aureus DNA in vitro, inducing an increase in absorbance of DNA samples at 260 nm due to the addition of Cecropin-XJ. In this study, with the increase in peptides concentration, the intensity of the maximum peak of DNA were increased, suggesting the double helix structure of DNA are destroyed by peptides. Moreover, the hyperchromicity induced by M-S is weaker than P W1 and thanatin, which is consistent with the results of gel retardation. In addition, the interaction was also examined by applying EB as an extrinsic fluorescence probe. The results suggested that M-S, thanatin, and P W1 can compete with EB in binding to DNA of *G. citri-aurantii* and some EB molecules intercalated into DNA base pairs were replaced by peptides. Moreover, the binding affinity of P W1 and thanatin was stronger than that of M-S. DAPI is a fluorescence dye which can bind with nucleic acid. Wang et al. found that the quantity of nucleic acid of S. aureus reduced after treated with soybean isoflavone for 28 h [25]. In this study, we also found that the DNA and RNA synthesis of *G. citri-aurantii* was also significantly reduced after treated with M-S, thanatin, and P W1 for 12 h. It indicated that M-S, thanatin, and P W1 can prevent nucleic acid synthesis of *G. citri-aurantii*, inducing a decrease in the nucleic acid content.

In the current study, the effect of antimicrobial M-S, thanatin, and P W1 on DNA of *G. citri-aurantii* was investigated, which laid the foundation for further study of the mechanism; however, the antimicrobial peptides are still some steps away for practical application due to the high cost of synthesis [35], and in the following research, it is possible for us to reduce the cost by performing heterologous expression or biosynthesis [36,37] of antimicrobial peptides to promote the large-scale application of antimicrobial peptides in controlling the postharvest disease of fruit and vegetable.

## 5. Conclusions

In summary, our results showed that M-S, thanatin, and P W1 were able to penetrate the cell membrane of *G. citri-aurantii* and gather in the cytoplasm. Nucleic acid may be one of the intracellular targets of the peptides. The results indicated that M-S, thanatin, and P W1 can bind to the *G. citri-aurantii* genomic DNA in vitro through electrostatic interaction, and intercalate into the base pairs in a helix of DNA, followed by wreck the double-helix structure. In addition, M-S, thanatin, and P W1 can also inhibit the synthesis of DNA and RNA of *G. citri-aurantii*.

## Data Availability

Data are contained within the article.
